# Supplementary material for: Comparative Impact of Organic Grass-Fed and Conventional Cattle-Feeding Systems on Beef and Human Postprandial Metabolomics—A Randomized Clinical Trial
Source: Metabolites. 2024 Oct 3;14(10):533. doi: 10.3390/metabo14100533 (PMC11509860; doi:10.3390/metabo14100533)
Supplement: Supplementary file 1 [file metabolites-14-00533-s001.zip › metabolites-3211450-supplementary.pdf]

metabolites

MDPI

Supplementary Materials

Comparative Impact of Organic Grass-Fed and Conventional Cattle-Feeding Systems on Beef and Human Postprandial Metabolomics—A Randomized Clinical Trial

Meghan Spears [1,†], Gwendolyn Cooper [2,†], Brett Sather [2], Marguerite Bailey [2], Jane A. Boles [3], Brian Bothner [2] and Mary P. Miles [1,*]

[1] Department of Food Systems, Nutrition, and Kinesiology, Montana State University, Bozeman, MT 59717, USA; meghanspears@montana.edu
[2] Department of Chemistry and Biochemistry, Montana State University, Bozeman, MT 59717, USA; gwendolyn.cooper@student.montana.edu (G.C.)
[3] Department of Animal and Range Sciences, Montana State University, Bozeman, MT 59717, USA
* Correspondence: mmiles@montana.edu; Tel.: +1-406-944-6678
† These authors contributed equally to this work.

Data Availability: Metabolomic Data: https://www.metabolomicsworkbench.org/.

Supplementary Figures

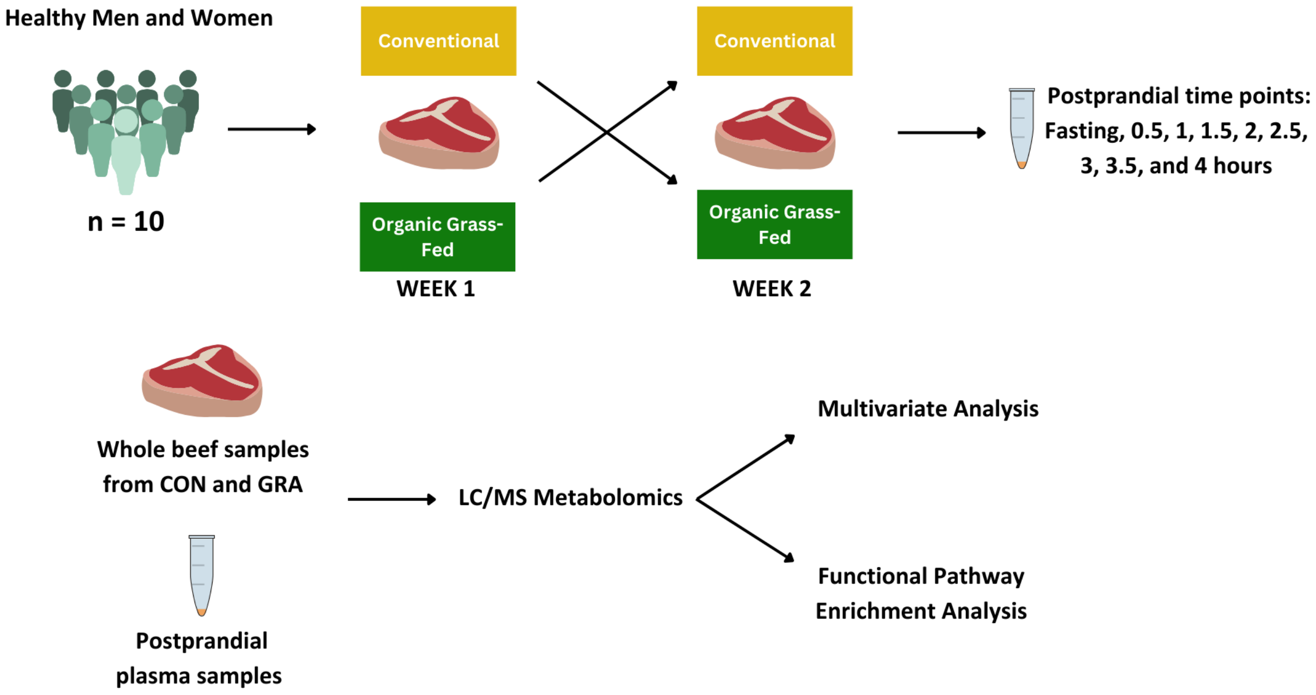

Figure S1. Double-blind, crossover study design overview.

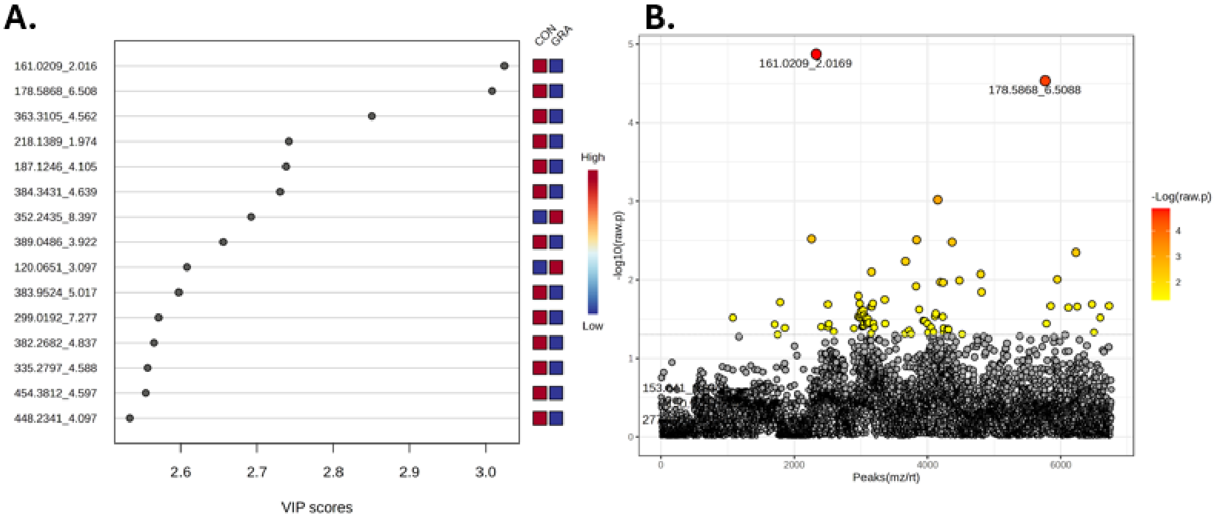

Figure S2. Variance Importance in Projection (VIP) and t-test plots for conventional versus grass-fed steak analysis. (**A**) VIP plot shows the scores of features that differentiate grass- versus conventional beef steaks. Larger VIP scores indicate a larger contribution of that variable to the model. Red indicates a higher abundance whereas blue indicates a lower abundance of the particular feature in the corresponding condition. (**B**) Two-sample t-test plot of conventional versus grass-fed steaks. Yellow, orange, and red circles represent 81 statistically significant features (raw p-value <0.05). Black circles represent 6679 features detected in the analysis that are statistically unsignificant.

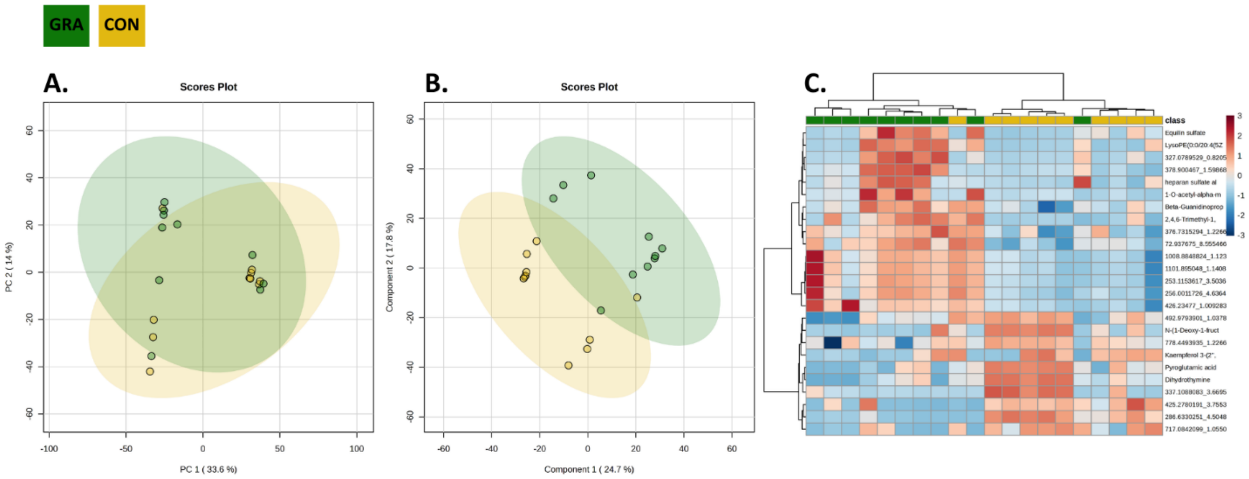

Figure S3. Fasting metabolomic profiles (**A**) PCA, (**B**) PLS-DA, and (**C**) heat map. All features were included in (**A**) and (**B**). The top 25 features were selected for (**C**).

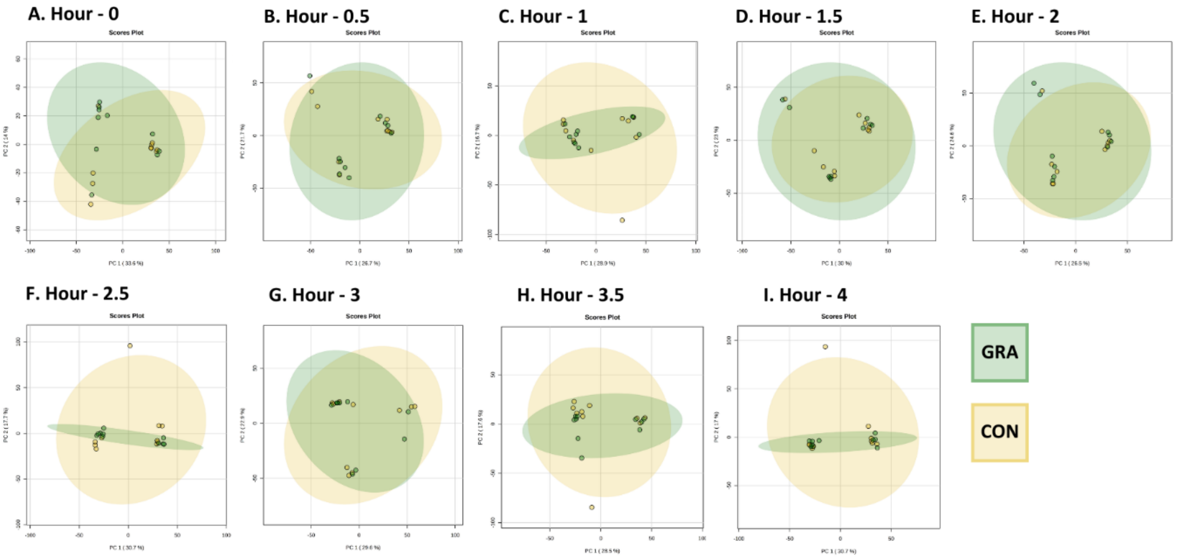

Figure S4. PCA of metabolic profiles at (**A**) fasting, (**B**) hour 0,5, (**C**) hour 1, (**D**) hour 1.5, (**E**) hour 2, (**F**) hour 2.5, (**G**) hour 3, (**H**) hour 3.5, and (**I**) hour 4.

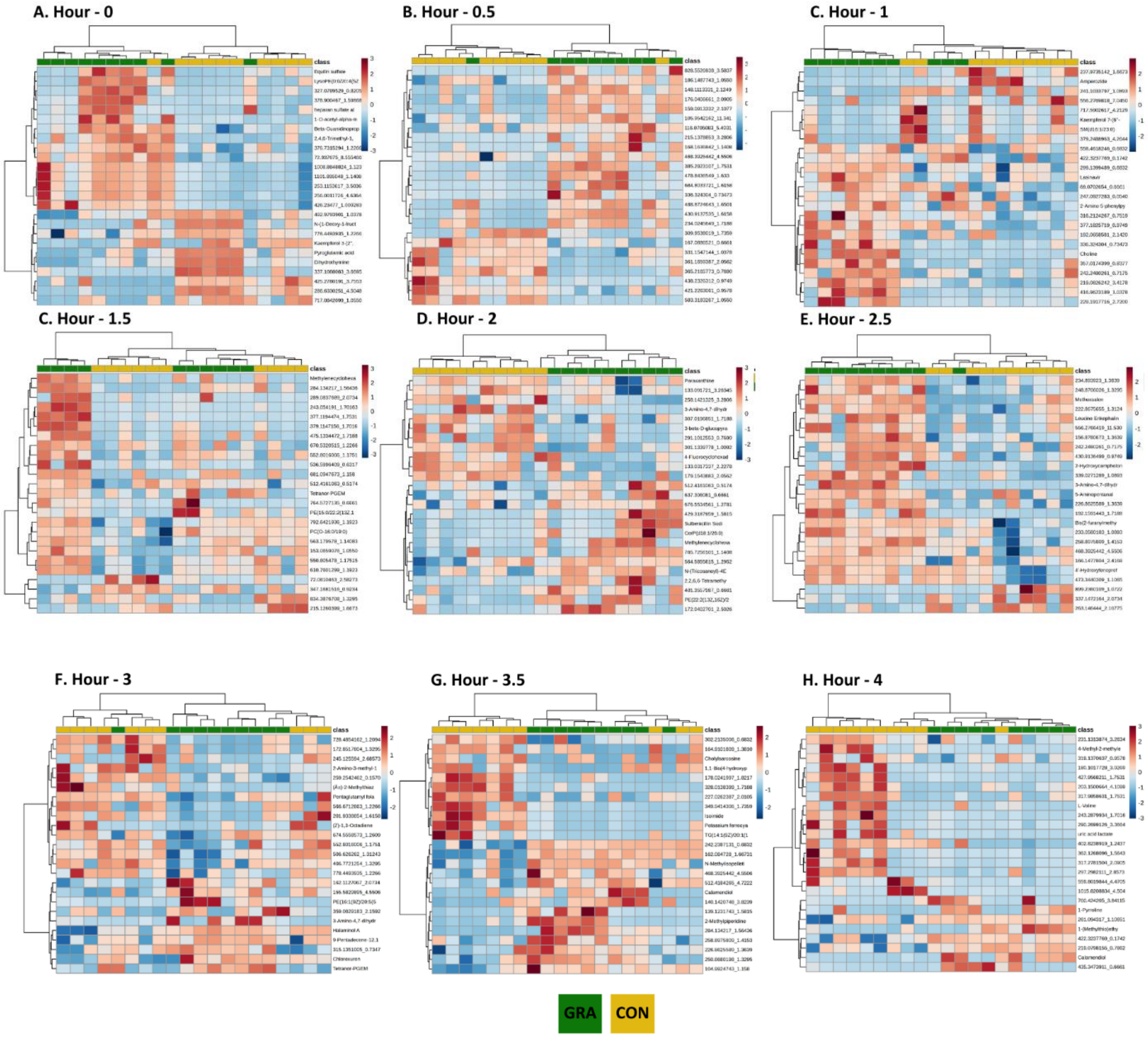

Figure S5. Heat maps of metabolic profiles score at (**A**) fasting, (**B**) hour 0,5, (**C**) hour 1, (**D**) hour 1.5, (**E**) hour 2, (**F**) hour 2.5, (**G**) hour 3, (**H**) hour 3.5, and (**I**) hour 4. Top 25 features are included in all figures.

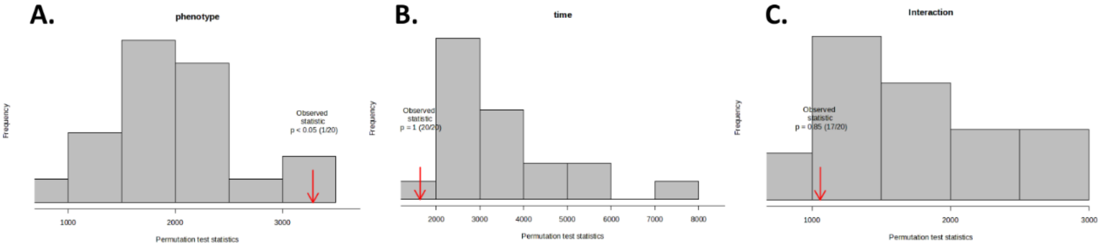

Figure S6. Model validation results through permutations of ANOVA simultaneous component analysis (ASCA) of phenotype (condition), time and the interaction of condition and time. Permutation times were set to 20 due to the size of the dataset. All features were included.

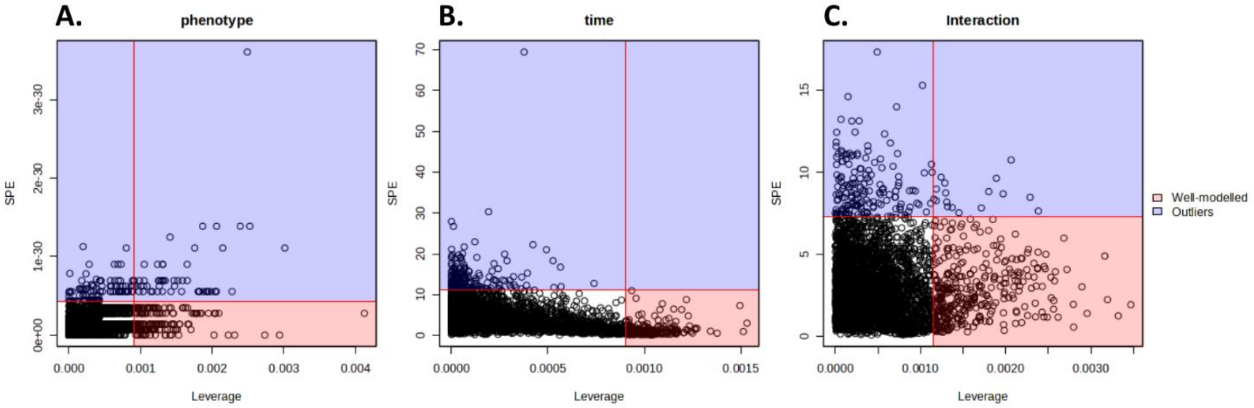

Figure S7. Model fitting plots of ASCA analysis of the effect of condition, time, and the interaction of these. Horizontal and vertical lines represent squared prediction error and leverage values. The alpha value cutoff was set to 0.05. The leverage threshold was set to 0.9. All features were included.

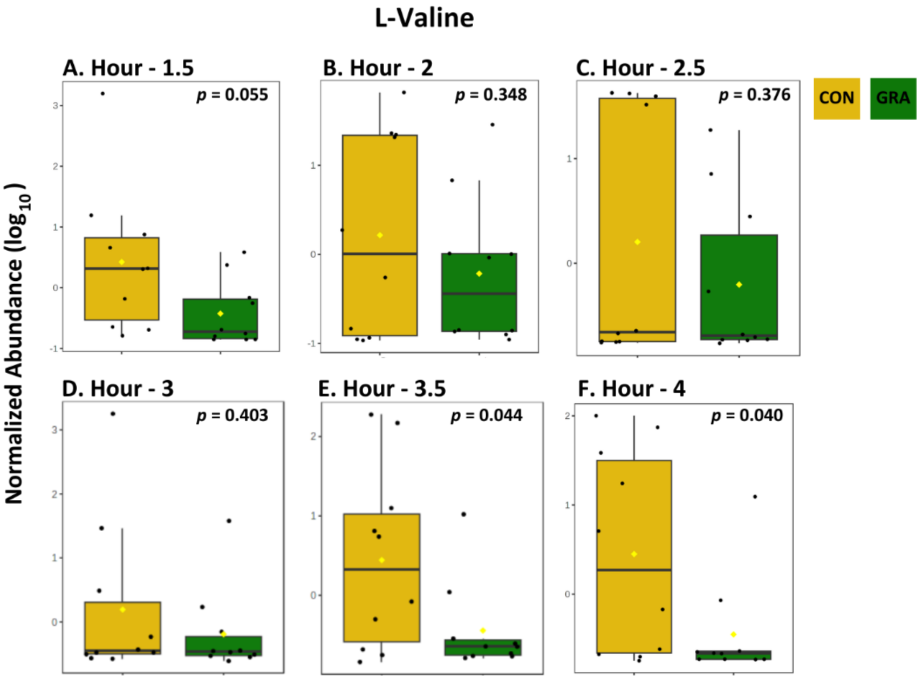

Figure S8. Box plots of the normalized abundance ($\log_{10}$) of L-Valine at postprandial timepoints (**A**) hour 1.5, (**B**) hour 2, (**C**) hour 2.5, (**D**) hour 3, (**E**) hour 3.5, and (**F**) hour 4 between conventional (yellow) and grass-fed conditions. P values are raw p values from paired t-test.

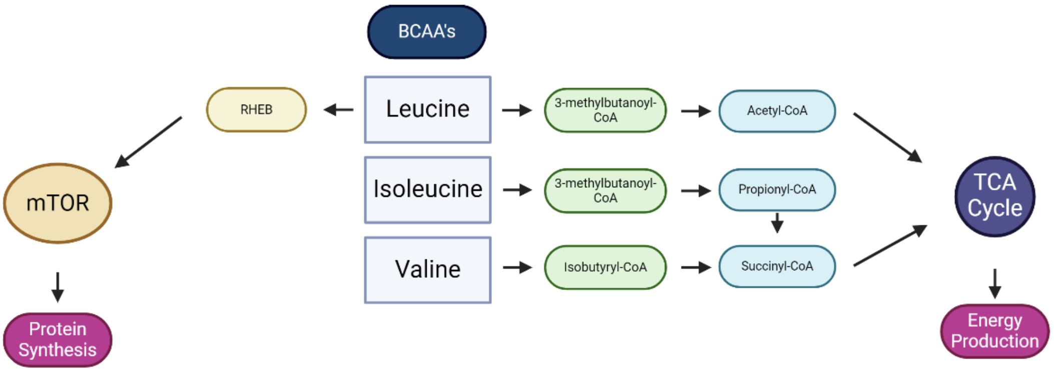

Figure S9. BCAA metabolism. BCAA's (valine, leucine, and isoleucine) play an important role in both energy production and protein synthesis. mTOR; mammalian target of rapamycin, RHEB; Ras homolog enriched in brain. Created with BioRender.com.

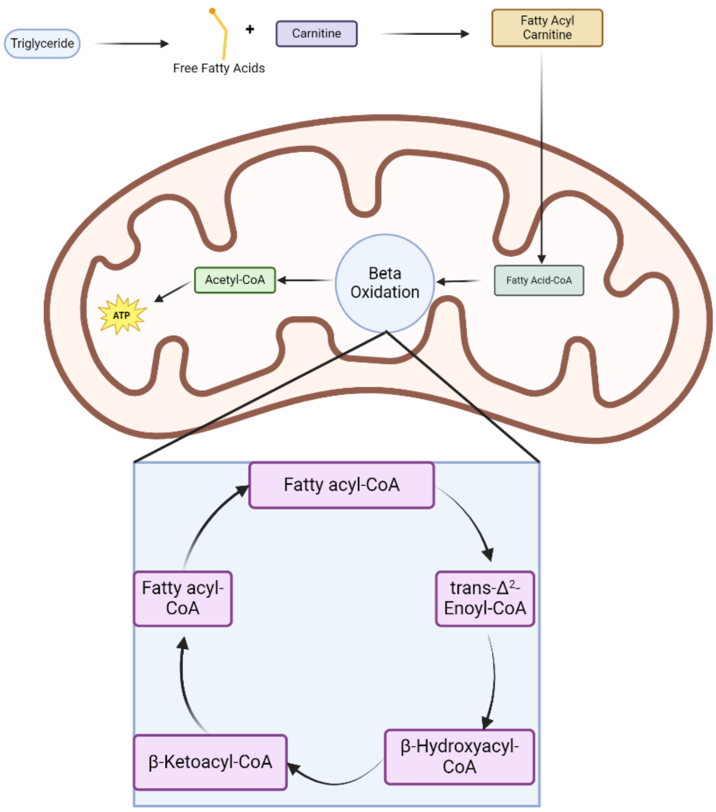

Figure S10. Carnitines and SFA oxidation. The role carnitines play in transportation of long-chain fatty acids into the mitochondria where they are oxidized to ultimately produce ATP. Fatty acyl carnitine enables the transport of fatty acids though the mitochondrial bilayer. Once within the mitochondria, beta oxidation occurs and ultimately results in the production of ATP. Inset represents beta-oxidation of SFA. Created with BioRender.com.
